# Total Body Irradiation without Chemotherapy as Conditioning for an Allogeneic Hematopoietic Cell Transplantation for Adult Acute Myeloid Leukemia

**DOI:** 10.1155/2016/1257679

**Published:** 2016-11-13

**Authors:** Sultan Altouri, Mitchell Sabloff, David Allan, Harry Atkins, Lothar Huebsch, Dawn Maze, Rajiv Samant, Christopher Bredeson

**Affiliations:** ^1^Division of Hematology, Department of Medicine, University of Ottawa, Ottawa, ON, Canada; ^2^Ottawa Hospital Research Institute, Ottawa, ON, Canada; ^3^Division of Radiation Oncology, The Ottawa Hospital Cancer Centre, University of Ottawa, Ottawa, ON, Canada

## Abstract

Current therapies for acute myeloid leukemia (AML), failing induction, are rarely effective. We report our experience in 4 patients with AML who received 16 Gy TBI prior to allogeneic hematopoietic cell transplantation (alloHCT), between June 2010 and May 2011. Patients were 20 to 55 years of age, 2 with relapsed disease and 2 with AML failing induction. An HLA-matched graft from related or unrelated donor was infused on day 0. All but one, who received a CD34^+^-selected graft, received methotrexate and tacrolimus +/− antithymocyte globulin, as GVHD prophylaxis. The other patient received tacrolimus alone. Neutrophil and platelet engraftment occurred at a median of 18 and 14 days, respectively. Patients were discharged at a median of 28 days. There were no unexpected toxicities in the first 30 days. One patient had cytomegalovirus (CMV) viremia and anorexia, at two months. One patient had grade 2 acute GVHD of the skin. One patient developed chronic GVHD of the eyes, mouth, skin, joints, and lung at 4 months. Two patients died from relapse of their leukemia at days 65 and 125. Two patients remain in remission beyond day 1500. 16 Gy TBI followed by an alloHCT for AML, failing induction, is feasible and tolerable.

## 1. Introduction

Acute myeloid leukemia (AML), failing induction chemotherapy, also referred to as refractory AML (rAML) has a very poor prognosis and may occur in up to 30% of the patients. Response to further cycles of chemotherapy rarely achieves a sustained remission. However, long-term remission may be achieved more frequently with an allogeneic hematopoietic cell transplantation (alloHCT), approaching an overall survival of 20–30% beyond 2 years [1–9]. More effective therapies for patients with rAML are needed in order to address this difficult problem [10, 11].

Intensification of the conditioning regimen represents a promising strategy to improve outcomes but has been limited by excessive transplant-related morbidity [12, 13]. Previous studies, over 20 years ago, suggested that there was an increased antileukemic effect using a higher dose of total body irradiation (TBI) in combination with cyclophosphamide (CY) (12 versus 15.75 Gy). However, this was overshadowed by toxicities, which resulted in a similar overall survival, establishing Cy/TBI (12 Gy) as the standard conditioning before transplantation [14].

Attempts at reducing the morbidity and mortality of the conditioning regimen have focused on developing safer and more effective components [7, 15–19]. Modern TBI delivery systems combined with improved techniques and better supportive care have permitted safe dose escalation of the conditioning regimen [20, 21]. Among these studies, a recent study demonstrated the safety of the use of fractionated TBI, as the sole component of the conditioning regimen, up to a dose of 20 Gy, in the autologous transplant setting for patients with non-Hodgkin's lymphoma [22]. It is timely to reconsider the tolerability of higher doses of TBI with the goal of improving outcomes for patients with rAML [23].

Herein, we report our experience using TBI (16 Gy) without chemotherapy as conditioning for alloHCT for rAML at the Ottawa Hospital, a regional tertiary care referral center. Patient characteristics and alloHCT data outcome are presented in Tables 1 and 2, respectively.

## 2. Case #1

A 34-year-old woman was diagnosed with AML, June 27, 2008. She was otherwise well. Cytogenetic analysis revealed a normal karyotype. She received induction chemotherapy with idarubicin 12 mg/m^2^ IV daily for three days and cytarabine 200 mg/m^2^ by continuous IV infusion daily for 7 days (IDAC). After achieving a complete remission (CR), she received another course of the same chemotherapy. Because of a slow recovery of her blood counts she did not receive any further consolidation. An alloHCT was considered but due to the fact that she did not have a suitable donor this was not pursued. She was followed at regular intervals in clinic until she relapsed, February 17, 2011, 923 days after achieving a CR. She received induction again with mitoxantrone, 10 mg/m^2^ IV daily for five days, and etoposide 100 mg/m^2^ IV daily for 5 days (NOVE). A repeat bone marrow aspiration did not demonstrate a CR, with 10–12% blasts remaining in the marrow. She then received high dose cytarabine, 3 g/m^2^ IV q12h, on days 1, 3, and 5 (HDAC). During this time an alloHCT was being planned to follow this cycle of chemotherapy. A bone marrow aspirate 2 days prior to the alloHCT demonstrated a CR. An alloHCT proceeded on May 19, 2011. The conditioning regimen, starting on day −3, consisted of TBI (16 Gy), delivered in 7 fractions (2.28 Gy/fraction) over 4 days, twice a day, using a system that has been previously described [24, 25], without any chemotherapy. TBI was delivered with 18 MV photons using a megavoltage linear accelerator at a dose rate of 120–140 cGy/min at midplane. There was a minimum of 6 hours between fractions. Custom-made lead lung and kidney attenuators were used on each field. The attenuator thickness (approximately 1.0 cm) was determined from a treatment planning CT scan and was designed to ensure that the lungs and kidneys received up to a maximum dose of 12 Gy. A 2.4 cm thick polymethyl methacrylate beam spoiler was placed above the patient and used to provide full skin dose. Graft versus host disease prophylaxis included tacrolimus, starting on day −3 (0.07 mg/Kg twice daily, adjusted to an AM serum concentration of 5–10 *μ*g/L) and methotrexate (administered on days 1 (15 mg/m^2^), 3, 6, and 11 (10 mg/m^2^, daily), following the alloHCT). Antithymocyte globulin (ATG) (Thymoglobulin, Sanofi-Aventis Canada Inc., Quebec, Canada) was administered on day −2 (0.5 mg/Kg) and day −1 (2 mg/Kg). Palifermin 60 *μ*g/Kg/day was also administered on days −6, −5, and −4. Bone marrow was infused on day 0 from a 7/8 unrelated donor (CD34^+^ cell dose: 4.3 × 10^6^/Kg). Neutrophils and platelets engrafted on days 29 and 15, respectively, and she was discharged on day 31. She did not have any evidence of acute or chronic graft versus host disease. She required narcotics for 8 days for mucositis and was on TPN for 21 days. She required IV home hydration for 2 weeks following discharge. At day 65 she was treated for cytomegalovirus (CMV) viremia with IV ganciclovir. This patient did not relapse and is alive beyond day 1500.

## 3. Case #2

A 20-year-old woman G3 P2 was diagnosed with AML, March 16, 2010, at 18 weeks of gestation. She was otherwise well. Cytogenetic analysis revealed a normal karyotype. After termination of the pregnancy she received induction chemotherapy, IDAC. Because she was not in remission she received a cycle of NOVE. The next cycle was changed to fludarabine 30 mg/m^2^ IV daily for five days, cytarabine 2 g/m^2^ IV daily for five days, and filgrastim 480 *μ*g sc daily, starting from the first day of chemotherapy until neutrophil count of 1 × 10^9^/L (FLAG) because she still did not achieve remission. During this time an alloHCT was being planned to follow this cycle of chemotherapy. At the time of the alloHCT she still had circulating blasts in the blood, indicating that she was not in CR. An alloHCT proceeded on August 12, 2010. The conditioning regimen, TBI 16 Gy, was as above. Graft versus host disease prophylaxis included tacrolimus, starting on day −1 without methotrexate or ATG. Palifermin was also administered as above. CD34^+^-selected product was infused on day 0 from a 7/8 related donor (CD34^+^ cell dose: 2.56 × 10^6^/Kg). Neutrophils and platelets engrafted on days 18 and 19, respectively, and she was discharged on day 32. She did not have any evidence of acute or chronic graft versus host disease. She required narcotics for 6 days for mucositis and was on TPN for 5 days. Evidence of disease recurrence appeared on day 35 and she died on day 65.

## 4. Case #3

A 55-year-old woman was diagnosed with AML, June 20, 2010. She was otherwise well. Cytogenetic analysis revealed an abnormal karyotype which included t(9;11). She received induction chemotherapy with IDAC. After achieving a complete remission (CR), she received another course of the same chemotherapy which was followed by a course of HDAC. An alloHCT was considered, but she declined it at this time. She was followed at regular intervals in clinic until she relapsed, January 19, 2011, 198 days after achieving a CR. She received induction again with NOVE. A repeat bone marrow aspiration did not demonstrate a CR, with 5–9% blasts remaining in the marrow. She then received FLAG. During this time an alloHCT was being planned to follow this cycle of chemotherapy. A bone marrow aspirate, 2 days prior to the alloHCT demonstrated a remission; however her platelet count was only 80, not quite meeting the definition of a CR. An alloHCT proceeded on May 27, 2011. The conditioning regimen, TBI 16 Gy, was as above. Graft versus host disease prophylaxis included tacrolimus, starting on day −2, methotrexate, and ATG, as above. Palifermin was also administered as above. Peripheral blood cells were infused on day 0 from a 8/8 unrelated donor (CD34^+^ cell dose: 11.38 × 10^6^/Kg). Neutrophils and platelets engrafted on days 16 and 11, respectively, and she was discharged on day 17. She did have evidence of acute graft versus host disease of the skin, grade 2, on day 32 which responded to prednisone. She did not have evidence of chronic graft versus host disease. She required narcotics for 7 days for mucositis. Evidence of disease recurrence appeared on day 75 and she died on day 125.

## 5. Case #4

A 25-year-old woman was diagnosed with AML, August 18, 2010. She was otherwise well. Cytogenetic analysis revealed an abnormal karyotype which included trisomy 8, 9, and 11. She received induction chemotherapy with IDAC. A repeat bone marrow aspiration did not demonstrate a CR, with 10–15% blasts remaining in the marrow. She then received NOVE. During this time an alloHCT was being planned to follow this cycle of chemotherapy. A bone marrow aspirate after this last cycle and 21 days prior to the alloHCT demonstrated a CR. An alloHCT proceeded on November 18, 2010. The conditioning regimen, TBI 16 Gy, was as above. Graft versus host disease prophylaxis included tacrolimus, starting on day −1 and methotrexate, as above. No ATG was used. Palifermin was also administered as above. Peripheral blood cells were infused on day 0 from a 8/8 related donor (CD34^+^ cell dose: 8.42 × 10^6^/Kg). Neutrophils and platelets engrafted on days 17 and 12, respectively, and she was discharged on day 26. She did not have evidence of acute graft versus host disease; however she did develop extensive chronic graft versus host disease with a maximum score of 3 involving the skin, liver, eyes, lungs, and vagina. This was initially steroid-refractory and required multiple lines of therapy, including imatinib, rituximab, and extracorporeal photopheresis. This has resulted in sclerosis of the skin and joint contractures. She required narcotics for 7 days for mucositis. This patient did not relapse and is alive beyond day 1500.

## 6. Discussion

In this report we describe our initial experience with TBI, as the sole component of the conditioning regimen of an alloHCT to treat rAML. Using 16 Gy in four patients we demonstrated that all of the early outcomes such as engraftment and time to discharge were within the expected limits without excessive morbidity. Toxicities beyond day 100 after alloHCT were limited to cGVHD in one patient with extensive disease. No deaths from the treatment were observed. 2/4 patients demonstrated long-term survival. Relapses, although likely recipient-derived, were not confirmed.

Higher doses of TBI or TBI as the sole component of the conditioning regimen for an alloHCT stem from studies published over 20 years ago describing a randomized study comparing CY/TBI (12 versus 15.75 Gy). Long-term follow-up from this randomized trial of two irradiation regimens for patients receiving alloHCT during first remission with AML demonstrated fewer relapses in the higher dose TBI group but an increased treatment related mortality, negating any benefit and establishing 12 Gy as the standard upper dose [13]. At that time TBI was delivered by opposing ^60^Co sources at a rate of 6-7 Gy/min daily for 6 (2 Gy/d) or 7 (2.25 Gy/d) days and each fraction took over an hour. The cause of death in these patients included relapse, aGVHD +/− infection or hemorrhage +/− CMV pneumonia +/− EBV lymphoma, sinusoidal obstructive syndrome of the liver, and graft failure with infection. Modern TBI has evolved being delivered through linear accelerators, using CT scanning to more accurately map out various areas and using attenuators to limit doses to critical structures like the kidney and lungs. Linear accelerators can deliver the dose more rapidly (i.e., 15 min versus 1-2 hours) minimizing the risk of nausea in the treatment room, potentially delaying treatment. Attenuators permit the delivery of more uniform and predictable doses. In addition, better antiemetics and supportive measures, including preemptive treatment for CMV, and multiple choices for the prophylaxis and treatment of GVHD are used, potentially improving the safety of a modern alloHCT [23, 26].

A number of studies over the past decade have tried to reexplore increased doses of TBI, in the modern era given the improvements in the delivery of TBI and supportive measures [20, 21, 23]. Usually it is in the context of higher doses of TBI with CY or other chemotherapies. Results have been mixed between 13.2 and 15.6 Gy with respect to toxicity at the upper end. Therefore by further modifying the conditioning regimen through the elimination of the chemotherapy component we are striving to maximize the radiation dose to realize its benefits, something that was not possible over 20 years ago.

Finally, a more recent study was done, based on 9 patients with lymphoma in the autologous transplant setting using TBI, alone, without any chemotherapy, at doses of 16, 18, and 20 Gy. They described that patients experienced the usual toxicities from TBI, specifically gastrointestinal toxicities, including nausea vomiting and mucositis. Although they may have been more severe and lasted longer than at the usual doses, they were all manageable and resolved. Long-term side effects were related to prolonged cytopenia and neuropathies. Once again, all were manageable and resolved over time. Only one patient died in this study, 429 days after transplant, of a gastric ulcer and its subsequent complications, which was believed to be secondary to prolonged steroid use, treating complications from the transplant. Overall, it was felt that by removing the CY, one was able to safely increase the dose of TBI [22]. Therefore, it appears that the dose of TBI can be safely increased above the usual 12 Gy under certain settings, if all attempts to minimize overlapping toxicities with the TBI are removed.

Raising the dose of TBI is further supported by in vitro data that demonstrated that there is a direct relationship between the dose of irradiation and the amount of leukemia that is killed, without any evidence of a plateau [27, 28]. In contrast, the chemotherapy portion of the conditioning has a narrow therapeutic window and evidence is building that it may be responsible for much of the toxicity, limiting its ability to be dose escalated [29]. Further such patients have already demonstrated their relative resistance to chemotherapy, all supporting the rational to raise the dose of TBI.

The two main limiting factors in this analysis are the retrospective nature and small population. However, the data was collected by members of the team treating these patients who were most familiar with them and most of it was extracted from the hospital computer system, avoiding the task of extracting data from paper and handwritten notes. Although the number of patients was small, this also permitted us to be meticulous in the data extraction, which can be more difficult when performing a retrospective analysis on a large number of patients.

Continuing efforts to improve outcomes of poor risk hematological diseases with myeloablative alloHCT through modifications in the conditioning regimen appear warranted. Intensification of the conditioning regimen, without increasing toxicity, may extend the therapeutic window for patients with aggressive leukemia. TBI (16 Gy) appears tolerable, in our small series of patients. Further dose escalation may be required to improve disease control. To explore this hypothesis, we are performing a prospective single arm trial using TBI (18 Gy) followed by alloHCT to treat patients with rAML.

## Figures and Tables

**Table 1:** Patient characteristics.

Patient number	Age (years) and gender	Cytogenetics	Timing of diagnosis (days prior to cell infusion)	Treatment #1 and response (days prior to cell infusion)	Treatment #2 and response (days prior to cell infusion)	Treatment #3 and response (days prior to cell infusion)	Relapse (days prior to cell infusion)	Treatment #1 and response (days prior to cell infusion)	Treatment #2 and response (days prior to cell infusion)
1	20, female	46 XX	Day −149	IDAC Refractory Day −146	NOVE Refractory Day −98	FLAG Refractory Day −48	NA	NA	NA

2	25, female	49 XX, +8, +9, +11	Day −92	IDAC Refractory Day −91	NOVE CR Day −57	NA	NA	NA	NA

3	37, female	46 XX	Day −1056	IDAC CR Day −1049	IDAC Continuing CR Day −1003	NA	Day −91	NOVE Refractory Day −83	HiDAC CR Day −38

4	55, female	46 XX, t(9,11)	Day −370	IDAC CR Day −366	IDAC Continuing CR Day −324	HiDAC Continuing CR Day −268	Day −128	NOVE Refractory Day −126	FLAG CR Day −58

IDAC: idarubicin 12 mg/m^2^ IV daily for three days and cytarabine 200 mg/m^2^ by continuous IV infusion daily for 7 days; NOVE: mitoxantrone, 10 mg/m^2^ IV daily for five days, and etoposide 100 mg/m^2^ IV daily for 5 days; FLAG: fludarabine 30 mg/m^2^ IV daily for five days, cytarabine 2 g/m^2^ IV daily for five days, and filgrastim 480 *μ*g sc daily, starting from the first day of chemotherapy until neutrophil count of 1 × 10^9^/L (FLAG); HDAC: cytarabine, 3 g/m^2^ IV q12h on days 1, 3, and 5.

**Table 2:** alloHCT data outcome.

Patient number	Disease status at time of alloHCT	Donor HLA type	Cell dose (CD34 × 10^6^/Kg)	GVHD prophylaxis	Outcome After alloHCT
1	Refractory	7/8 MRD	2.56	Tacrolimus	Relapse Day +35
					Died of disease progression Day +65

2	CR	8/8 MRD	8.42	Tacrolimus and MTX	Alive in CR Day +1354

3	CR	7/8 MRD	4.3	Tacrolimus and MTX	Alive in CR Day +1145

4	CR	8/8 MUD	11.38	Tacrolimus and MTX	Relapse Day +75
					Died of disease progression Day +125

CR: complete remission, HLA: human leukocyte antigen, MRD: matched related donor, MUD: matched unrelated donor, MTX: methotrexate, GVHD: graft versus host disease, alloHCT: allogeneic hematopoietic cell transplantation.

## Appendix

### A. Definitions

Neutrophil and platelet engraftment were defined as the first of three and seven consecutive days with a sustained absolute neutrophil and platelet count of > 0.5 × 10^9^/L and > 20 × 10^9^/L in the absence of platelet transfusions, following alloHCT, respectively. Adverse events were recorded according to the National Cancer Institute Common Terminology Criteria for Adverse Events (version 4.0) and Bearman toxicity grading scale [4]. GVHD scoring used the modified Seattle Glucksberg criteria for acute GVHD (aGVHD) and the National Institute of Health consensus criteria for chronic GVHD (cGVHD) [12, 27].

### B. Supportive Care

All patients received care according to current clinical practice for transplant recipients as outlined in the policies and procedures of the Ottawa Hospital Blood and Marrow Transplant Programme (TOHBMT), which is an accredited program of the Foundation for the Accreditation of Cellular Therapy (FACT).

#### B.1. Supportive Medication

Allopurinol: 300 mg PO daily for the first 5 days.
Ondansetron: 8 mg PO or IV q12h starting prior to the first fraction of TBI and continuing until 48 hours after the last fraction of TBI.
Ursodiol: 250 mg PO tid starting prior to the first fraction of TBI and continuing for 30 days following the alloHCT.
Acyclovir: 400 mg PO bid or 200 mg IV bid, starting on day −4 and continuing until discharge from the transplant hospital admission; acyclovir 800 mg PO bid administered starting at the time of hospital discharge and continuing until 12 months after the transplant.
Fluconazole: 400 mg PO daily starting on day −4 and continuing until the absolute neutrophil count (ANC) is greater than 0.5 × 10^9^/L.
Trimethoprim/sulfamethoxazole: two single strength tablets given twice weekly on Monday and Thursday to all patients after the neutrophil count was above 0.75 × l0^9^/L and continuing for 6 months following transplantation.
Ceftriaxone: 1 g IV daily starting once the neutrophil or the white cell count drops below 0.5 or 1.0 × l0^9^/L, respectively.
Piperacillin-tazobactam: initiated immediately for a fever > 38°C in the presence of ANC < 0.5 × l0^9^/L or at any time that clinical signs of infection are present.
Palifermin: 60 *μ*g/kg/day IV for 3 days prior to the start of radiation, administered at the discretion of the attending physician.
Fentanyl transdermal and dilaudid: used for pain control.

#### B.2. Nutritional Support

Oral hygiene was maintained by saline mouthwashes. Patients unable to eat or swallow received enteral nutrition via a nasogastric feeding tube until a sustained oral intake of at least 1500 kcal/day was achieved. If nasogastric feeding was not tolerated, total parenteral nutrition (TPN) was administered for nutritional support.

#### B.3. Blood Product Support

UV-irradiated, leukoreduced, packed red blood cells and platelet concentrates were used for all transfusions. Two units of packed red blood cells were given if the morning hemoglobin was less than 80 g/L. Platelet support was given prophylactically if the morning platelet count was below 10–20 × 10^9^/L.
